# Conditioning to Enhance the Effects of Repetitive Transcranial Magnetic Stimulation on Experimental Pain in Healthy Volunteers

**DOI:** 10.3389/fpsyt.2022.768288

**Published:** 2022-02-22

**Authors:** Léa Proulx-Bégin, Alberto Herrero Babiloni, Sabrina Bouferguene, Mathieu Roy, Gilles J. Lavigne, Caroline Arbour, Louis De Beaumont

**Affiliations:** ^1^Department of Psychology, Université de Montréal, Montreal, QC, Canada; ^2^Centre de recherche du CIUSSS du Nord-de-l'Île-de-Montréal, Montreal, QC, Canada; ^3^Division of Experimental Medicine, McGill University, Montreal, QC, Canada; ^4^Faculty of Dentistry, McGill University, Montreal, QC, Canada; ^5^Department of Psychology, McGill University, Montreal, QC, Canada; ^6^Faculty of Dental Medicine, Université de Montréal, Montreal, QC, Canada; ^7^Faculty of Nursing, Université de Montréal, Montreal, QC, Canada; ^8^Department of Surgery, Faculty of Medicine, Université de Montréal, Montreal, QC, Canada

**Keywords:** transcranial magnetic stimulation, therapeutics, placebo effect, conditioning, psychological, pain, hypoalgesia

## Abstract

**Objective:**

In this proof-of-concept study we sought to explore whether the combination of conditioning procedure based on a surreptitious reduction of a noxious stimulus (SRPS) could enhance rTMS hypoalgesic effects [i.e., increase heat pain threshold (HPT)] and augment intervention expectations in a healthy population.

**Methods:**

Forty-two healthy volunteers (19–35 years old) were enrolled in a randomized crossover-controlled study and were assigned to one of two groups: (1) SRPS and (2) No SRPS. Each participant received two consecutive sessions of active or sham rTMS over the M1 area of the right hand on two visits (1) active, (2) sham rTMS separated by at least one-week interval. HPT and the temperature needed to elicit moderate heat pain were measured before and after each rTMS intervention on the right forearm. In the SRPS group, conditioning consisted of deliberately decreasing thermode temperature by 3°C following intervention before reassessing HPT, while thermode temperature was held constant in the No SRPS group. Intervention expectations were measured before each rTMS session.

**Results:**

SRPS conditioning procedure did not enhance hypoalgesic effects of rTMS intervention, neither did it modify intervention expectations. Baseline increases in HPT were found on the subsequent intervention session, suggesting variability of this measure over time, habituation or a possible “novelty effect.”

**Conclusion:**

Using a SRPS procedure in healthy volunteers did not enhance rTMS modulating effects on experimental pain sensation (i.e., HPT). Future studies are therefore needed to come up with a conditioning procedure which allows significant enhancement of rTMS pain modulating effects in healthy volunteers.

## Introduction

Chronic pain is often characterized by the presence of abnormal sensory perception (1–3), manifested among others by decreased pain thresholds when they are measured by quantitative sensory testing (QST) methods (4, 5). QST is considered a valuable tool to assess the function of the somatosensory system, being useful not only to characterize pain conditions but also to evaluate treatment responses in clinical and healthy populations (4–7). In addition, post-intervention QST changes among healthy individuals have also proved to be useful in characterizing physiological pathways as well as discerning potential mechanisms of action (4, 7, 8), therefore “*bridging the gap”* between the identification of novel intervention strategies and the optimization of their efficacy (9, 10).

High frequency repeated transcranial magnetic stimulation (rTMS) is a non-invasive brain stimulation technique that was shown effective in increasing pain thresholds and inducing analgesia in different clinical populations, especially when applied over the primary motor cortex (M1) (11–14). Although the mechanisms underlying rTMS sensory modulating effects are not fully elucidated, they are thought to rely on the local activation of top-down processes in addition to involving widespread endogenous pain modulatory systems (15–18).

In that way, increases in thermal pain thresholds derived from QST measures were found following M1 rTMS relative to a sham intervention (19–25). However, results from sham-controlled studies are rather inconsistent and heterogeneous, with a high variability in treatment effects across the literature (14, 25, 26). One possibility to explain discrepancies among study results is the documented variable response to TMS techniques, participants often being categorized as responders and non-responders (27, 28). While it is possible that TMS responsiveness relies on connectivity and excitability patterns (29, 30), action mechanisms are not fully understood, especially in the pain field. Therefore, the understanding and investigation of strategies aiming to enhance rTMS analgesic effects are clinically relevant, as it could potentiate rTMS therapy success.

Like any other pain treatment, the sensory modulating effects of rTMS are thought to be due to the treatment itself combined with other non-specific effects, including placebo or expectations of the therapy being effective (31, 32). Indeed, the improvement of pain treatment therapies by increasing placebo effects has raised recent interest among the pain research community (33–35). Different methods have been suggested to enhance placebo effects, such as shaping and adapting information about analgesic treatments and/or associating the treatment with a positive context or response (36). While verbal suggestions are an easily implementable way to improve analgesic responses, it has been shown that prior positive therapeutic experiences could have more robust effects and better predict placebo response than verbal expectation ratings (37–39). One way to achieve such positive experience is to use conditioning paradigms, where medically connoted procedures (conditioned stimulus) are coupled to a pain stimulus (unconditioned stimulus), in which the intensity is surreptitiously reduced from baseline levels (40–42). Indeed, previous studies suggest that conditioning procedures can lead to longer-lasting effects and more significant placebo hypoalgesia when compared to methods such as verbal suggestion (40, 43, 44).

Here, we tested whether the rTMS hypoalgesic response could be enhanced by the use of a conditioning paradigm based on a surreptitious reduction of a noxious stimulus (abbreviated as SRPS by our team) induced with heat. We therefore conducted a proof-of-concept study using SRPS to modulate heat pain thresholds among healthy individuals, who were enrolled in a two-visit, twice-daily session rTMS protocol using parameters proven effective to increase thermal pain thresholds (23). In this protocol, active rTMS and sham interventions served as the conditioned stimulus and were coupled to experimental heat pain (i.e., unconditioned stimulus), in which the intensity was surreptitiously reduced or maintained depending on group assignment. Secondarily, we assessed if perceived expectations of intervention success could contribute to the hypoalgesic effects of rTMS and/or conditioning.

## Materials and Methods

The study was conducted in accordance with the Helsinki Declaration and approved by the Research Ethics Committee of the CIUSSS du Nord-de-l'Île-de-Montréal in Canada (Approval number: 2018-1525). All participants provided written informed consent and received monetary compensation.

### Participants

Forty-two healthy volunteers were successfully recruited through advertisements placed at the Université de Montréal's campus and in social media, and all procedures were performed in a TMS laboratory located at the Hôpital du Sacré-Coeur de Montréal. Criteria for exclusion were: (1) drug or alcohol abuse, (2) epilepsy, (3) metal implants/coils/electronic devices above the waist, (4) pregnancy, (5) psychiatric disorders, (6) chronic pain, and (7) inability to understand instructions. All subjects were naïve to any form of motor cortex stimulation. Aside from contraceptive pills, no medication or caffeine was allowed on the day of testing. All testing sessions took place in the morning to control for diurnal variations of cortical excitability (45, 46). Participants were told that the study aimed to investigate the effects of rTMS on experimental pain. To further avoid bias, participants were blinded to the nature of the assignment groups (i.e., that there were two types of interventions (active rTMS and sham) and were not initially informed that there was a possible conditioning procedure. Reasons for the latter incomplete disclosure and group assignment were revealed to participants by one investigator (LPB) during a debriefing session conducted after having completed the experimental protocol.

### Experimental Design

**Figure 1:**
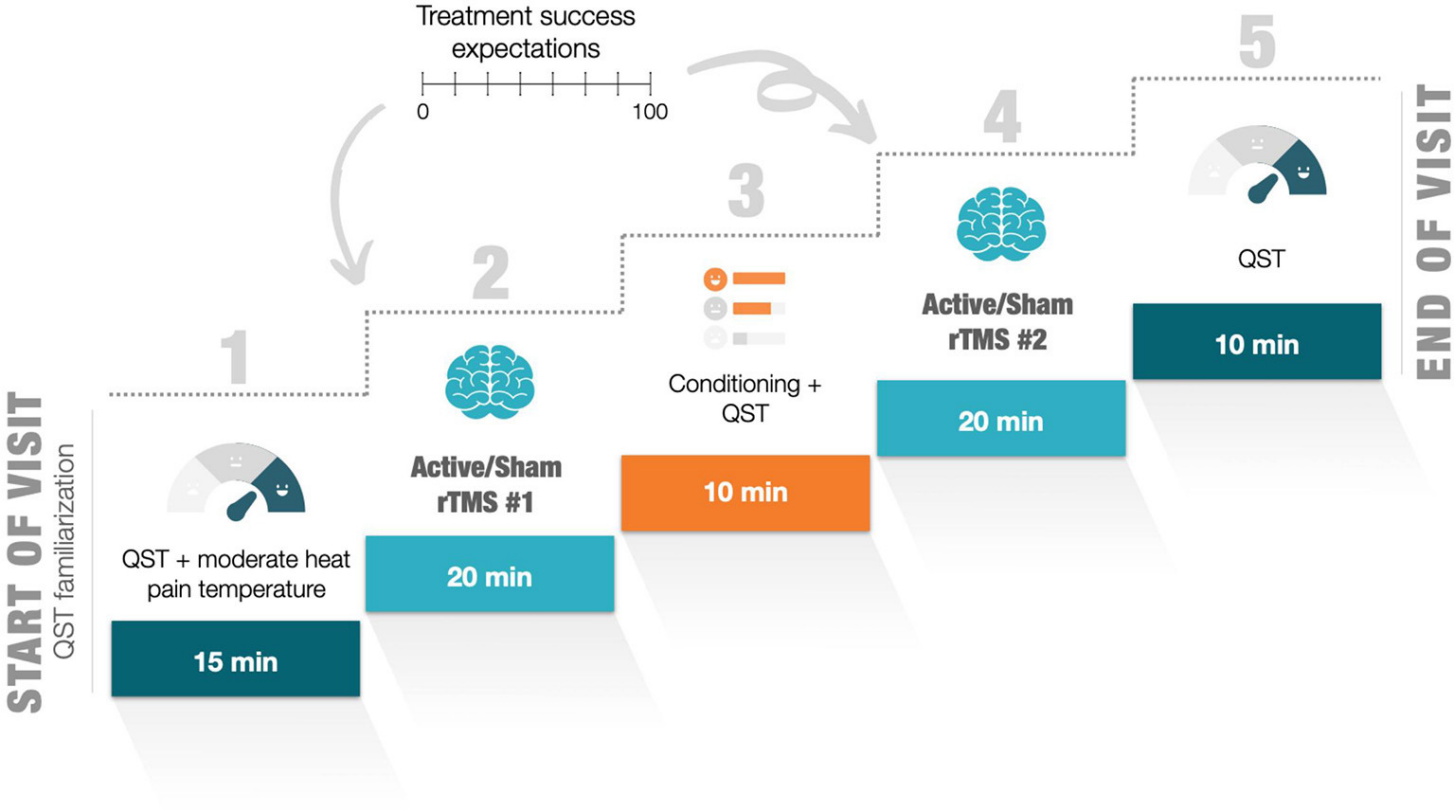
Outline of the experimental procedure for each visit. QST, quantitative sensory testing; rTMS, repetitive transcranial magnetic stimulation; VAS, visual analog scale.

A randomized crossover-controlled study design was implemented. After their inclusion, participants were randomly assigned to one of two groups: (1) SRPS and (2) No SRPS. In spite of their group allocation, each participant took part in two single-day laboratory visits, one with active rTMS and the other with a sham intervention, separated by at least 1 week to avoid any potential carry-over effects of the first visit on the other (22, 47, 48). Each visit included two consecutive sessions of rTMS (or sham) spaced 10 min apart (Figure 1). Heat pain threshold (HPT) was measured at three different time points, namely before, between and after each rTMS/sham session. Moreover, perceived expectations of intervention success were also assessed before each rTMS/sham session.

### Main Outcomes Measures

The main outcome of this study was participants' HPT, which was assessed at three different time points [1**—**baseline (pre-rTMS/-sham); 2**—**post-rTMS#1/-sham#1; 3**—**post-rTMS#2/-sham#2] across groups (SRPS, no SRPS) and intervention types (rTMS or sham). The secondary outcome was perceived expectations of intervention success, assessed prior to and following each intervention in both groups.

### Randomization, Concealment, and Blinding

The order of the interventions (rTMS or sham at first or second visit) and the group assignation (SRPS or no SRPS) were randomized and counterbalanced using a computer-based random sequence generation program (https://www.random.org/lists/). The randomization procedure was carried out by an external member of the research group and consisted of 42 sealed, opaque and numbered envelopes that contained information about group assignment and intervention order. When a participant was recruited, another staff member not involved in the study used the randomization list to determine which envelope was assigned to the participant and then forwarded the respective information to the QST experimenter (assignation group) and to the assistant in charge of setting the rTMS parameters (type of intervention), who was different than the TMS operator. Participants and TMS operator were therefore blinded to group assignment and intervention. Only the TMS assistant knew about the intervention administered, adjusting stimulus parameters and coil used (active/sham) accordingly while the TMS operator and participant were outside the room. Moreover, the experimenter in charge of sensory testing and expectation assessments was unaware of the type of intervention. Experimenters were all women, and their role did not vary throughout the study. They also wore a white lab coat and provided scripted neutral instructions.

### Questionnaires

On the first visit, participants completed a series of questionnaires to assess sociodemographic and psychosocial characteristics known to potentially interfere with pain sensitivity (49–53), such as the Beck Depression Inventory-II (BDI-II) (54), the State-Trait Anxiety Inventory (STAI) (55), the Pain Catastrophizing Scale (PCS) (56), the Perceived Stress Scale (PSS) (57), and the Pittsburgh Sleep Quality Index (PSQI) (58).

### Quantitative Sensory Testing

#### Heat Pain Threshold

Noxious heat was induced using the Medoc Pathway Pain and Sensory Evaluation System (Medoc TSA 2001-II, Ltd, Israel) operating according to the principles of the Peltier effect with a 3 cm^2^ thermode.

At the beginning of each visit, participants were seated in a quiet room held at a constant temperature (22 °C) where they were trained before the formal HPT testing on a different area of the ventral forearm than the one used for the testing, in order to familiarize them with the procedure (unrecorded data). This training was conducted in both visits to ensure accuracy and reproducibility of the tests throughout the experiment. Assessment of the HPT was determined according to the “method of limits” (4).

From a baseline temperature of 32 °C, heat thermal stimulations were applied at 5 cm from the right wrist flexion crease with a linear rate of 1 °C/s. Participants received three successive stimuli of increasing heat with inter-stimulus intervals of 30 s in order to prevent pain habituation or temporal summation of pain. Participants were asked to press on a button when they detected the first perception of pain up to 49 °C, to prevent tissue damage. The average temperature over three trials was calculated for the determination of HPT. Given the nature of the study, we focused our thermal procedures on HPT, which is thought to have better intra- and inter-rater reliability and less variability over time relative to other QST measures, to avoid as much as possible confounding effects of time between visits (59, 60). Moreover, since our SRPS procedure is based on heat, we thought that HTP was the most adequate outcome to assess intervention changes.

#### Conditioning Procedure Using SRPS

To determine the individualized temperature needed to elicit moderate heat pain, a sequence of successive phasic heat pain stimuli between 41 and 49°C separated by 30s intervals was administered at 10 cm from the right wrist flexion crease (ventral fore arm), with a starting baseline temperature of 32° C, incremental rate of 4° C/s, and a 7 s plateau (61). After each stimulation, participants' pain intensity was evaluated on a 0-100 visual analog scale (VAS: 0 corresponding to “no pain” and 100 to “the worst pain imaginable”) in order to find the temperature corresponding to participants' moderate pain intensity. Moderate pain intensity was considered the lowest temperature corresponding to a value of 40–60/100 on the VAS (62). The determined temperature was applied once again after the first intervention in participants assigned to the no SRPS group, while a conditioning manipulation, consisting of deliberately decreasing by 3 °C the determined temperature, was performed with patients assigned to the SRPS group. The conditioning manipulation was carried out to induce a positive experience of hypoalgesia prior to the next intervention. The group without SRPS was exposed to the same temperature prior to the second intervention. To ensure that the 3 °C decrease was sufficient to induce a positive experience of hypoalgesia in the participants, a VAS measurement was performed after exposure to the conditioned (or not) temperature.

### rTMS and Sham Intervention

#### Identification of Simulation Site and Resting Motor Threshold

At the first visit, optimal stimulation site over the left M1 was determined through exploration near the C3 cortical electrode site as per the 10/20 International system of electrode placement (63). The optimal stimulation position was determined as the stimulation site which elicited the largest and most consistent motor evoked potentials (MEPs) recorded from the contralateral first dorsal interosseous muscle. The “hot-spot” was marked on a swim cap with a dermatograph pencil to allow accurate repositioning of the coil between intervention and throughout the whole experiment. The angle of inclination of the coil was determined using a level and the distance between the bathing cap and the nasion and between the bathing cap and each earlobe were also measured. The resting motor threshold (rMT) was defined as the lowest stimulator output needed to induce a MEP of >50 μV peak-to-peak amplitude in at least 6/10 consecutive trials (64). Once the rMT was determined, the experimenter in charge of the rTMS administration and the participant left the rTMS room while waiting for the TMS assistant to set stimulation modalities and coil used, the sham coil being visually identical and emitting similar sounds during stimulation than the active coil. Prior to each intervention session, participants' expectations of intervention success were measured given that it could influence intervention response (65, 66). Thus, participants were asked: “How useful do you think non-invasive stimulation techniques such as rTMS can be in reducing pain?” and instructed to respond with a 0–100 VAS scale (i.e., 0 corresponding to “these techniques are not useful” and 100 to “these techniques are very useful”).

#### Intervention Protocol

The rTMS treatment consisted of a series of 20 trains of 6 s duration (54 s intertrain interval) at a stimulation rate of 10 Hz and at an intensity corresponding to 80% of the rMT (1,200 total pulses) (11, 25). rTMS was applied over the left M1 using the Magstim Double 70 mm AirFilm® Coil (Magstim, Whitland, Wales, UK). The TMS coil was positioned tangentially to the head at a 45° angle to induce a posterior-anterior current flow (12). The coil was centered and fixed directly over the stimulus site using a tripod so that the coil handle pointed to the back. Sham treatment was applied using the same procedure with the Magstim AirFilm® SHAM coil (Magstim, Whitland, Wales, UK).

### Debriefing

At the end of the study, a debriefing session was conducted with participants to reveal the true nature of the study. Then, participants were asked to guess their assignment group and the order they received the sham or rTMS (first or second visit). Afterwards, the group assignment and intervention order were revealed to participants by the investigator (LPB). Participants completed a new consent form to obtain their agreement to retain their data.

### Statistical Analyses

Statistical analyses were performed using IBM SPSS Statistics software version 25 (Armonk, NY, United States). A Shapiro-Wilks test was used to ensure that HPT measures and expectations data were normally distributed. Parametric tests were performed with a statistical significance set as *P* ≤ 0.05. Descriptive analyses were also used to characterize and compare all groups on various demographic data. Results are expressed as means, standard deviation (SD) and percentages. Independent-sample Student's *t*-tests were performed for continuous socio-demographic data (i.e., the questionnaires) and Chi-squared tests were used for nominal data such as the sex and the blinding efficacy measure. In order to assess our main objective, a three-way mixed analysis of variance (ANOVA) was conducted to examine the effects of different interventions (rTMS vs. sham), *time points* (baseline, post-rTMS/-sham#1, post-rTMS/-sham#2), and *groups* (SRPS vs. no SRPS) on the modulation of HPT. Secondarily, a three-way mixed ANOVA was also computed to evaluate the effects of groups (SRPS vs. no SRPS) and times points (baseline, post-rTMS/-sham#1) and interventions (rTMS vs. sham) on expectations of intervention success. Greenhouse-Geisser corrections were used for the two ANOVAs. If a significant interaction was obtained, we conducted *post-hoc* analyses and corrected for multiple comparisons using the Bonferroni test by adjusting the *p*-value according to the number of comparisons (*p* = 0.017). Main effects were interpreted only if interactions were not significant. Partial eta squared ($\eta_{\text{p}}^{2}$) are reported. Lastly, to ensure the effectiveness of our conditioning procedure, we calculated the difference on the VAS measure between pre-post conditioning measurement and then, two independent-sample Student's *t*-tests were computed, one for each intervention (rTMS, sham), to determine if there were differences in the VAS between the groups (SRPS, no SRPS).

As this study was a proof-of-concept in nature, no power calculation was carried out a priori. However, our sample size is comparable to other studies with similar objectives that were deemed to be adequately powered (24, 25).

## Results

### Demographic Information

Forty-two healthy participants were recruited for this proof-of-concept study. Of those, one participant was excluded due to severe depression symptoms as revealed with the Beck Depression Inventory scale, for a final data set of 41 right-handed healthy adults (20 females, 23.98 ± 3.16 years). Included participants were divided into two groups: SRPS group (*n* = 21; 10 females) and no SRPS group (n = 20; 10 females). Demographic information can be found in Table 1. Student's t-tests revealed no significant differences between groups (*p* > 0.05) on socio-demographic data except for perceived sleep quality during the last month (*p* = 0.035). However, this difference was considered anecdotal and not clinically significant given its low magnitude, the nature of the study population and the debated cut-off score for sleep disturbance using the PSQI in non-clinical samples (67).

**Table 1:** Demographic and clinical characteristics of the study sample.

**Variables**	**SRPS**	**No SRPS**	***p***
	**(*n = 21*)**	**(*n = 20*)**	
Sex (male/female)	11/10	10/10	0.883
Age (years)	23.76 (2.68)	24.20 (3.67)	0.664
Education (years)	16.00 (2.98)	16.10 (2.83)	0.913
Body mass index	23.39 (3.35)	24.19 (3.17)	0.431
Beck depression inventory (BDI-II)	3.76 (4.39)	4.15 (3.25)	0.750
Trait-anxiety (STAI-T)	33.29 (6.51)	35.20 (9.48)	0.454
State-anxiety (STA-T)	29.76 (5.33)	31.05 (6.23)	0.480
Pain catastrophization scale (PSC)	13.14 (7.74)	12.00 (8.07)	0.646
Perceived Stress Scale (PSS)	10.86 (5.40)	12.35 (7.37)	0.462
Pittsburgh Sleep Quality Index (PSQI)	3.67 (2.06)	5.20 (2.44)	0.035*
rMT—rTMS visit	64.43 (11.91)	66.70 (13.25)	0.567
rMT—sham visit	63.71 (14.04)	65.70 (12.15)	0.632

*Values are given as the mean (SD) or frequency (N = 41).*

*rTMS, repetitive transcranial magnetic stimulation; rMT, resting motor threshold; SD, standard deviation; SRPS, surreptitious reduction of pain stimulus*.

### Fluctuations in Heat Pain Threshold

**Figure 2:**
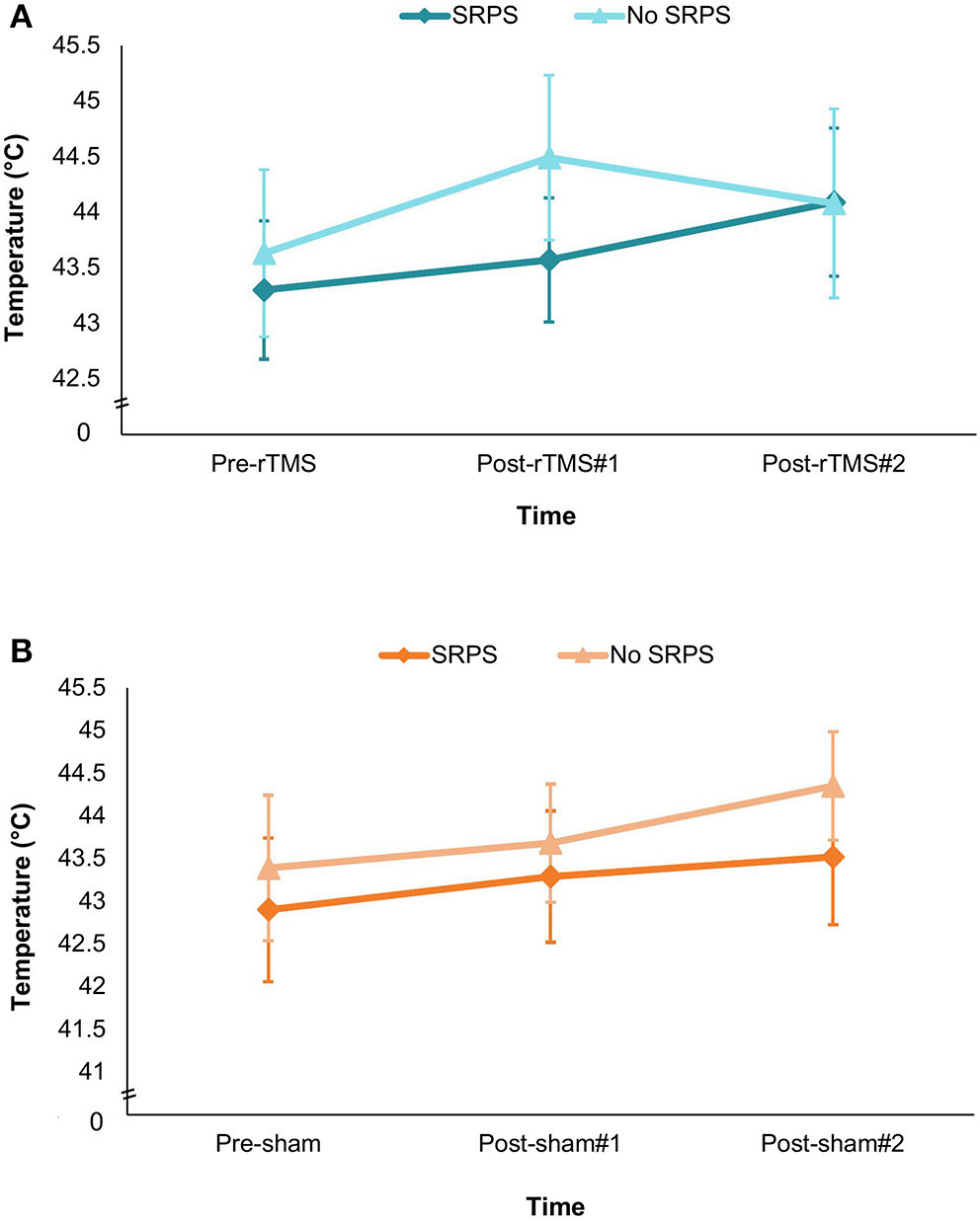
Graph depicting fluctuations in heat pain threshold (mean T°C) according to group × time (pre-interventions, between interventions and post-interventions) × SRPS exposition. **(A)** rTMS visit. **(B)** Sham visit. Results are expressed as means and standard errors (SEM). HPT, heat pain threshold; SRPS, surreptitious reduction of pain stimulus.

There was no significant interaction between the three factors (groups, intervention and time) for the HPT measure, *F*_(1.837, 39)_ =1.127, *p* = 0.33, $\eta_{\text{p}}^{2}$ = 0.028 (see Figure 2). In addition, none of the two-way interactions were significant. However, we found a significant main effect of time, *F*_(1.781, 39)_ = 5.493, *p* = 0.008, $\eta_{\text{p}}^{2}$ = 0.123. *Post-hoc* multiple comparisons analyses showed that HPT measures significantly differed between baseline and post-rTMS/-sham#2 time points (*p* = 0.005), while other comparisons (baseline vs. post-rTMS/-sham#1, *p* = 0.051; post-rTMS/-sham#1 vs. post-rTMS/-sham#2, *p* = 0.149) did not reach statistical significance. Descriptive statistics suggest that participants, regardless of the group or intervention received, tended to show an increase in HPT from baseline (*M* = 43.298 ± 2.953) to post-rTMS/-sham#2 (*M* = 44.008 ± 3.124) measures.

We also computed a paired-sample t-test to assess between-visit baseline HPT measure changes regardless of conditioning groups. We found a statistically significant between-visit HPT measure difference at baseline *t*_(40)_ = −4.299, *p* < 0.001. Descriptive statistics showed that on average, HPT threshold had increased by 2.0 °C at the second visit (*M* = 44.30 ± 2.71) relative to the first visit (*M* = 42.30 ± 3.82) (95% CI, −2.950 to −1.063) highlighting a higher baseline heat pain threshold at the second visit.

### Expectations

**Figure 3:**
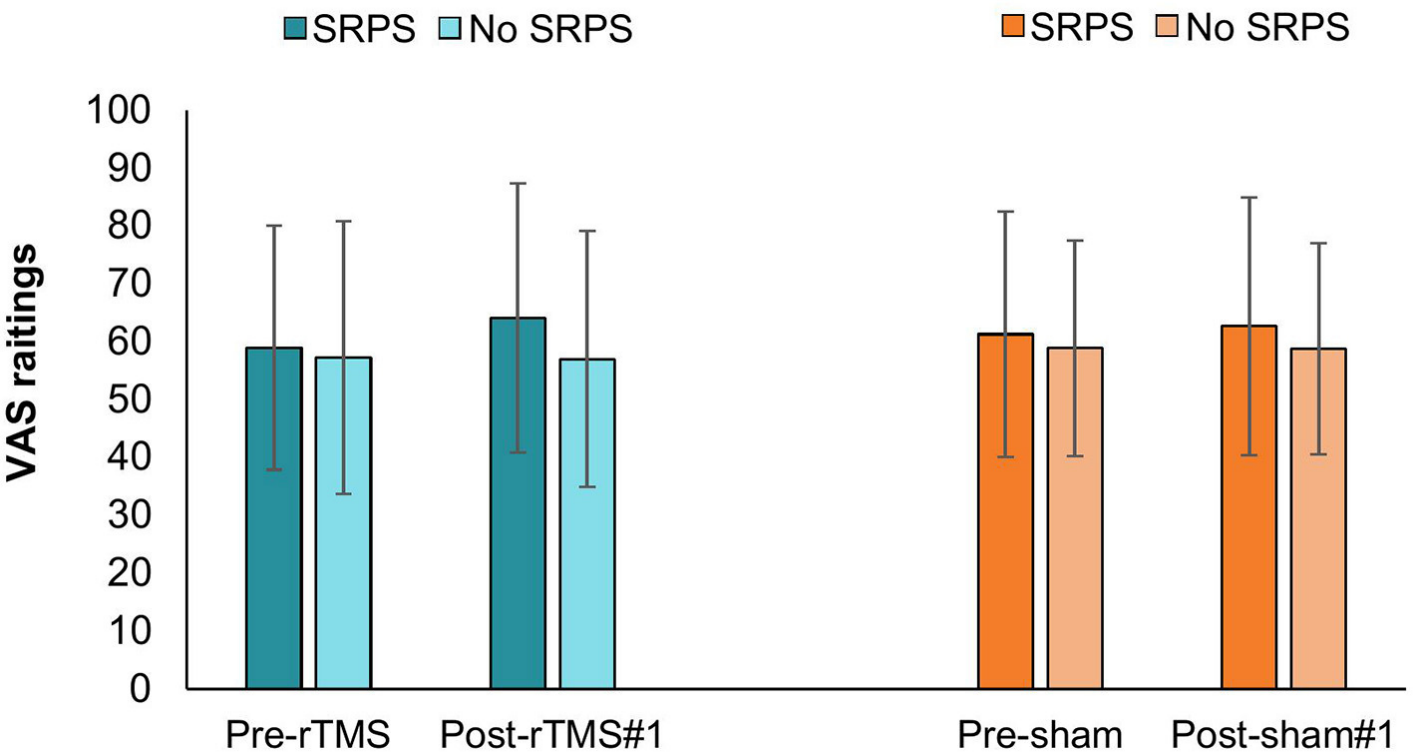
Fluctuations in VAS expectation ratings according to time × SRPS exposition during rTMS and sham visit. rTMS, repetitive transcranial magnetic stimulation; SRPS, surreptitious reduction of pain stimulus; VAS, visual analog scale.

The Groups^*^Time^*^Interventions on expectations was not statistically significant *F*_[1, 38]_ = 1.269, *p* = 0.27, $\eta_{\text{p}}^{2}$ = 0.032. Likewise, two-way interactions were not statistically significant, and no main effect was observed (*p* > 0.05) (see Figure 3).

### Positive Analgesic Experience Induction

**Figure 4:**
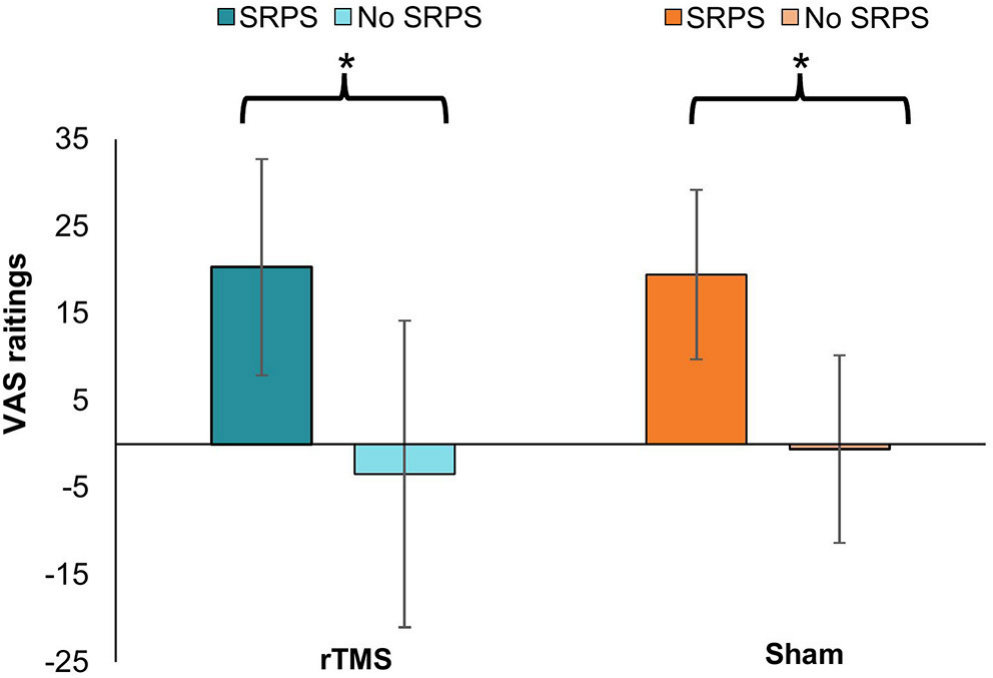
Differences from baseline VAS pain ratings according to SRPS exposure during rTMS and sham visit. rTMS, repetitive transcranial magnetic stimulation; SRPS, surreptitious reduction of pain stimulus; VAS, visual analog scale. The * indicates the difference between the groups (*p* ≤ 0.001).

The data distribution of the VAS values measured before the conditioning procedure respects the normality criteria proposed by Curran et al. (68) so that no data transformation had to be performed. Student's t-test showed that the conditioning procedure significantly reduced pain perception derived from the VAS measure relative to the no SRPS group, whether participants underwent the active rTMS intervention [*t*_(39)_ = −6.794, *p* ≤ 0.001] or the sham intervention [*t*_(39)_ = −4.371, *p* ≤ 0.001], indicating that decreasing by 3 °C the thermode temperature was sufficient to induce a detectable change in temperature perception (see Figure 4).

### Blinding Efficacy

While 20 participants (48.78%) correctly identified group assignation, 6 participants (14.63%) guessed it wrong, and 15 participants (36.59%) were unable to provide an answer. A Chi-square test revealed that these results were not statistically different (χ^2^ = 4.512, *p* = 0.11) across conditioning groups. Regarding the intervention order identification, 14 participants (34.15%) correctly distinguished the intervention order, 5 participants (12.19%) guessed it wrong, and 22 participants (53.66%) were not confident about the intervention order. The Chi-square test showed no significant difference between groups (χ^2^ =3.476, *p* = 0.18), suggesting a successful participant blinding.

### Adverse Effects

A significant between-group difference (χ^2^ = 9.466, *p* = 0.009) was found regarding adverse effects. While no participants in the no SRPS group reported any adverse effect during or following the interventions, 1 participant reported a mild and transient headache. Moreover, 33.33% (*n* = 7) participants in the SRPS group reported tingling sensations during the active rTMS intervention. No adverse effects were reported for the QST procedure.

## Discussion

The results of this study indicate that combining a SRPS conditioning paradigm to rTMS did not significantly enhance analgesic response to noxious heat over the forearm nor intervention expectations among healthy individuals when compared to those not receiving conditioning. Moreover, prior exposure to HPT equivalently increased post-intervention HPT across conditioning or intervention types. Similarly, in spite of experimental condition blinding, we observed a modest increase in baseline HPT between Visit 1 and Visit 2, which may reflect normal variability of HPT over time as pointed out in other studies (69, 70), but also a possible “novelty effect” on Visit 1.

The induction of placebo effects could represent a low-risk and cost-effective strategy in order to potentiate treatment response to pain stimuli and an important bulk of research has been building over the years in this regard (71). Placebo effects are complex phenomena involving several brain networks and psychophysiological mechanisms, such as the endogenous opioid, endocannabinoid, oxytocin, vasopressin, and dopamine systems (31, 72). Studies have suggested the involvement of several action mechanisms based on different theories and models, such as conditioning and expectancy, which could be potentially manipulated to optimize therapeutic approaches and ultimately outcomes (33, 36, 73). For instance, it has been shown that improving patients' preoperative expectations and placebo effects was associated with fewer days of hospitalization and better long-term outcomes in patients undergoing cardiac surgery (74, 75) and reduced opioid intake after spine surgery (76). Moreover, a meta-analysis including 27 studies revealed medium to large effects of verbal suggestion, conditioning (paired with verbal suggestion), and mental imagery on experimental and acute procedural pain and small effects on chronic pain (77). In parallel, studies have shown that experimental manipulations aiming to pre-conditioning individuals with effective analgesic treatments, such as reducing the intensity of painful stimulation surreptitiously in order to make the subjects believe that analgesic treatments are effective, can induce a previous positive experience to the treatments and consequently improve placebo analgesia (37, 39, 61). This type of pre-conditioning is typically performed with topical analgesic interventions such as creams, ointments, injections, acupuncture, and oral pharmacologically (39, 43, 44, 78, 79), which are often more “accessible,” and thus individuals are expected to have prior experience with them. In contrast, prior exposure to rTMS intervention is very unlikely due to its limited accessibility, such that associated placebo effects and its possible manipulation to enhance analgesic experiences are less understood (80).

Treatment effects of active rTMS interventions are frequently compared to “sham” procedures, where an inactive coil with limited power, usually identical in aspect and producing similar noises than the active coil is used. The analgesic response to rTMS is heterogeneous across studies, especially when compared to sham stimulation (12–14). For example, a study showed that the effectiveness of a HF-rTMS protocol was easier to demonstrate against other active stimulation method than against a sham treatment (81). This has been partially attributed to the quality of the studies, including low sample sizes, lack of adequate randomization, and lack or poor blinding (12).

Growing awareness and media attention for non-invasive brain stimulation techniques and sophistication of setups and equipment, including sham coils, have been proposed as possible explanations (80). Additionally, another study revealed that the amount of placebo analgesia observed in a sham rTMS session depended on the success of a previous active rTMS response in neuropathic pain patients (82). In that study, there was no significant difference between the effects of the active and sham rTMS when the latter was applied after a successful rTMS session (82). Simply put, sham rTMS sessions induced significant analgesia (comparable to active rTMS) when they followed a successful rTMS rather than an unsuccessful rTMS, which could at least in part be the result of unconscious conditioned learning. The authors went on to discuss the importance of the timing of placebo relative to active interventions in rTMS studies for pain relief (82).

In the present study, we did not observe a significant intervention effect between SRPS and no SRPS groups (Figure 2). Moreover, the interaction between intervention (active/sham), time (baseline, between, and post measures) and group (SRPS/no SRPS) on HPT was not significant. A possible explanation might be related to our conditioning procedure. Previous literature has shown that expectations play an important role in the placebo response in experimental pain models and clinical populations (32, 35, 83). In our study, although the conditioning procedure was successful in inducing a positive analgesic experience (Figure 4), it did not seem to modulate participants' expectations (Figure 3) (84). We decided to use VAS 40/100 as a threshold of moderate or significant pain (i.e., minimal level of pain affecting performance in daily living) based on previous literature (62, 84), prior pilot data (unpublished), and also ethical issues (e.g., avoid severe levels of pain and/or disturbance). However, it is possible that higher VAS (e.g., 60/100) could have facilitated the perception of decreased pain after lowering thermode temperature in the SRPS group, thereby accentuating the placebo effect (37, 85). Whereas previous studies using SRPS performed a decrease of 2° C from the pain-inducing temperature (86), we decided to decrease pain-inducing temperature by 3 °C so as to make the SRPS more noticeable, yet believable. Nonetheless, some of our SRPS participants (n=2) did not experience any analgesic response after the conditioning, suggesting a possible nocebo effect after the first intervention (active or sham) due to anxiety for example, or a lack of understanding of the study instructions. Although speculative, one may question whether decreasing thermode temperature by a few more degrees could have modulated intervention response. Although future research is warranted, it is also plausible that combining conditioning and explicit verbal suggestions could have induced larger placebo effects (77).

Other possible explanation for the lack of difference between active and sham interventions could be related to the rTMS protocol modalities, including targeted location, frequency, intensity and number of sessions. It is recognized that high frequency stimulations over M1 present more consistent and analgesic effects when compared to other locations. However, stimulations over other locations such as the dorsolateral prefrontal cortex (DLPFC) have also shown analgesic properties in experimental and clinical pain (11, 87). Indeed, a single session study with similar rTMS parameters and design to the present study showed that active rTMS over both M1 and DLPFC similarly increased thermal pain thresholds (heat and cold) among healthy volunteers, suggesting comparable effects of DLPFC and M1 when compared to sham (24). In addition, there is also evidence showing analgesic and sensory modulatory effects of rTMS when applied to the primary or secondary somatosensory cortex (S1 and S2 respectively) (88, 89). In fact, one study favored rTMS stimulations over S2 relative to M1, DLPFC and sham in order to increase heat pain thresholds (90). However, locating optimal stimulation site over S2 depends on neuroimaging and neuronavigation methods, which complicates their implementation. Other important parameters of stimulation are frequency and intensity. Importantly, a study including 65 healthy participants undergoing QST pre- and post-rTMS stimulations (1Hz 80% resting motor threshold [Rmt], 1Hz 100%rMT, 10Hz 80%rMT, 10Hz 100%rMT, 50Hz triplets at 90% of active motor threshold) and sham over M1, revealed that protocols with higher frequencies had increased modulatory effects across several QST measures (23), which supports the use of our protocol. However, no main effects for TMS device parameters nor significant interaction effects were found for on HPT, which is similar to the results in our study. Moreover, effects of rTMS on QST measures were relatively small and variable across all rTMS conditions, suggesting that rTMS analgesic effects using laboratory-induced pain among healthy individuals may be difficult to discern. A possible reason is the presence of a ceiling effect, given that the somatosensory system of healthy individuals is thought to be normal and there is a limit for its enhancement, contrary to chronic pain patients where dysfunction and maladaptive networks can provide a more extensive range of modulation (i.e., chronic pain patients typically exhibit much lower HPT than healthy controls) (25).

In addition, it is known that a higher number of rTMS sessions usually yield larger analgesic effects (11, 13, 91, 92). Yet, one and two sessions involving similar rTMS protocols than the one used in the present study have been found to increase pain thresholds in healthy volunteers (25). One cannot exclude the possibility that additional rTMS sessions and perhaps conditioning sessions (i.e., increase of conditioning strength) could have resulted in larger increases in heat pain thresholds.

An important issue that was also observed in a recently published transcranial direct current stimulation (tDCS) study (69) was the high variability in baseline HPT from one visit to the other. In our study, we considered the potential confounding effects of several variables documented to influence the somatosensory system at baseline such as anxiety, depression, sleep, perceived stress, pain catastrophizing, and limited others at both visits such as medication and caffeine intake, circadian effects on QST and cortical excitability by performing both visits at the same time. We nonetheless observed a significant difference between baseline HPT values from visit 1 to visit 2 across both groups, as heat pain thresholds at baseline in visit 2 were considerably higher than at visit 1 regardless of intervention order, which might have limited potential intervention-related improvement at visit 2. As noted by Kold and Graven-Nielsen (69), it is possible that the decreased heat sensitivity at the second visit could be due to some kind of habituation to the sensory testing, and perhaps to the intervention. As participants previously been exposed several times to rTMS and QST during the prior visit, the novelty and salience could have decreased, which may have increased mind wandering, reduce attention and thus decrease sensory experience (93). Importantly, this did not appear to be influenced by an unsuccessful blinding, as most of the patients did not distinguish effectively between active or sham interventions.

Although this study presents with important methodological strengths, it is not without limitations. Firstly, the sample size may not have been sufficient to detect significant effects by groups and types of intervention. Secondly, the use of a cross-over design to assess intervention effects on measures from one visit to the other is susceptible to the possible variability of HPT over time [Wasner (70), #2996], making challenging to interpret the true effect of the treatment. While HPT are thought to be a reliable measure (59), longitudinal studies using repeated measures across more days may provide better understanding of QST day-to-day variability. Furthermore, cross-over designs usually carry learning effects that are difficult to control, which may have consequently confounded the results of sequential trials (94). Thirdly, this study was designed to serve as a proof-of-concept and it is based on experimental pain, which is used as a proxy for clinical pain. However, comparisons between experimental pain and clinical are often inconclusive, to say the least (95). Indeed, both rTMS analgesic responses and placebo analgesic effects have been shown to be higher among chronic pain populations (25, 96), which raises the possibility that replicating this study with clinical populations may yield different results. Investigating the determinants of rTMS analgesic response is an exciting research avenue that could benefit from the understanding and optimization of placebo effects.

## Conclusion

In conclusion, these results showed that the combination of a conditioning paradigm with rTMS was not effective to increase the analgesic response to experimental heat pain nor to enhance expectations with two sessions of rTMS among healthy individuals. Although the findings of this study were not significant, the observed results are still relevant to the TMS and placebo literature, as they are indicative of the challenges that this area of research may entail among experimental pain models with healthy participants. However, considering that chronic pain populations might present higher expectations for treatment efficacy and be more sensitive to conditioning and placebo effects, the use of conditioning to raise expectations and rTMS response deserves to be investigated further in chronic pain patients.

## Data Availability Statement

The raw data supporting the conclusions of this article will be made available by the authors, without undue reservation.

## Ethics Statement

The studies involving human participants were reviewed and approved by Hospital Sacre-Coeur of Montreal. The participants provided their written informed consent to participate in this study.

## Author Contributions

LP-B, AH, CA, and LD contributed to conception and design of the study. LP-B and AH organized the database and wrote the first draft of the manuscript. LP-B, SB, and LD performed the statistical analysis. All authors contributed to manuscript revision, read, and approved the submitted version.

## Funding

This work was supported by the Quebec Bio-Imaging Network Research (Grant Number: PP 16.04, 2018) and the Canada Research Chair in Pain, Sleep and Trauma of Dr. Gilles Lavigne (Grant Number: 950-229397).

## Conflict of Interest

The authors declare that the research was conducted in the absence of any commercial or financial relationships that could be construed as a potential conflict of interest.

## Publisher's Note

All claims expressed in this article are solely those of the authors and do not necessarily represent those of their affiliated organizations, or those of the publisher, the editors and the reviewers. Any product that may be evaluated in this article, or claim that may be made by its manufacturer, is not guaranteed or endorsed by the publisher.
